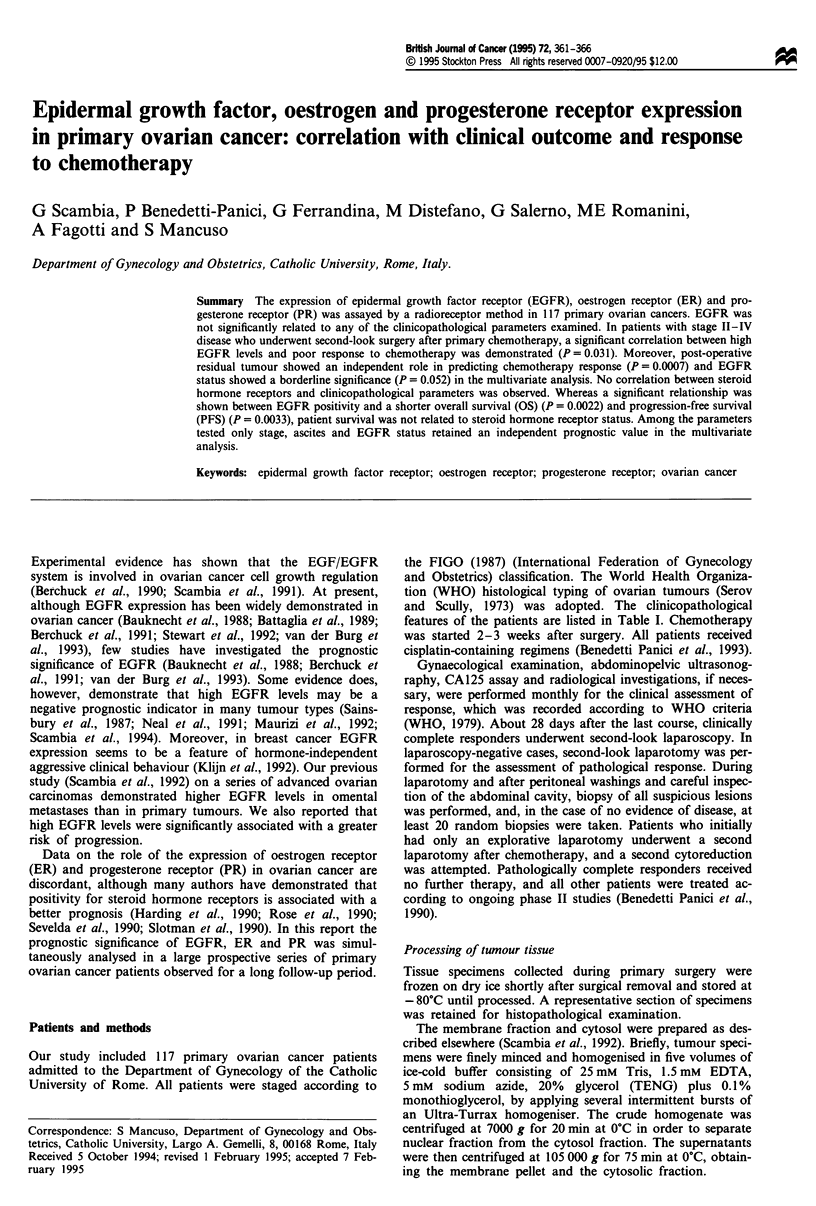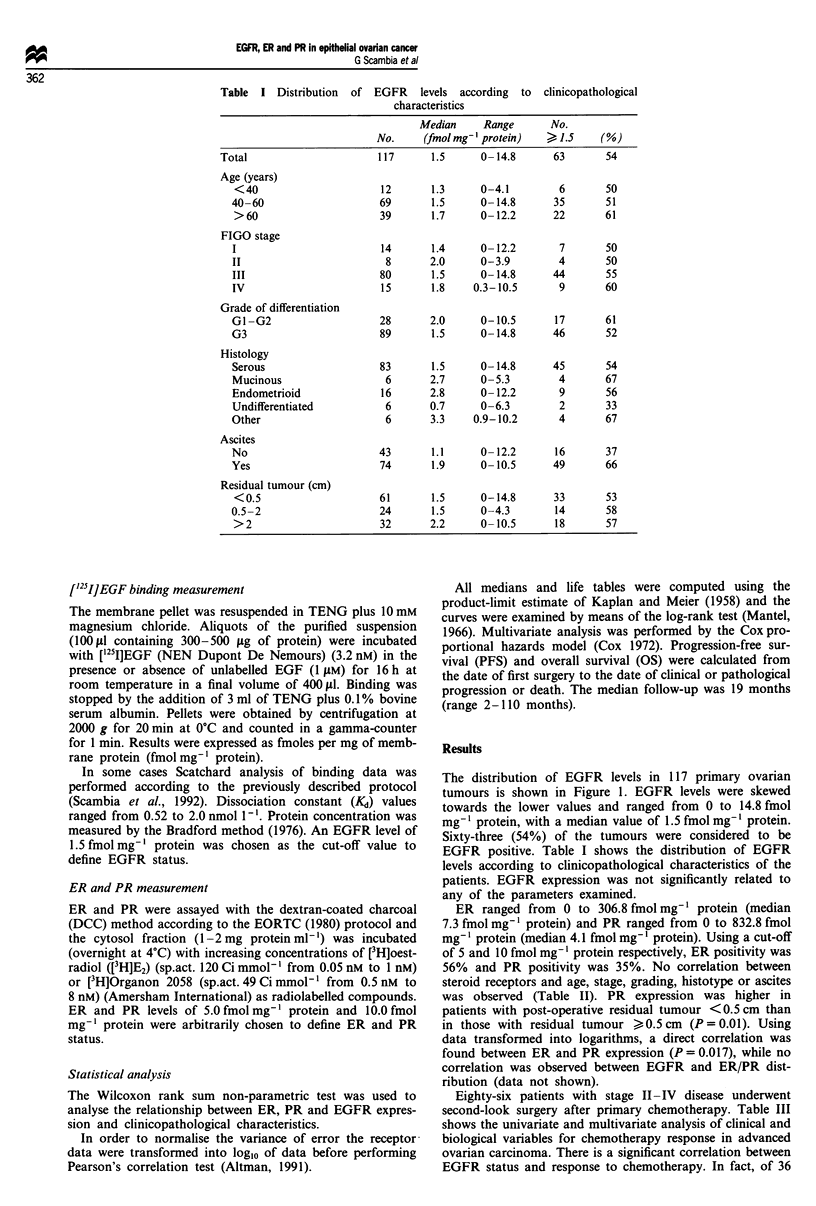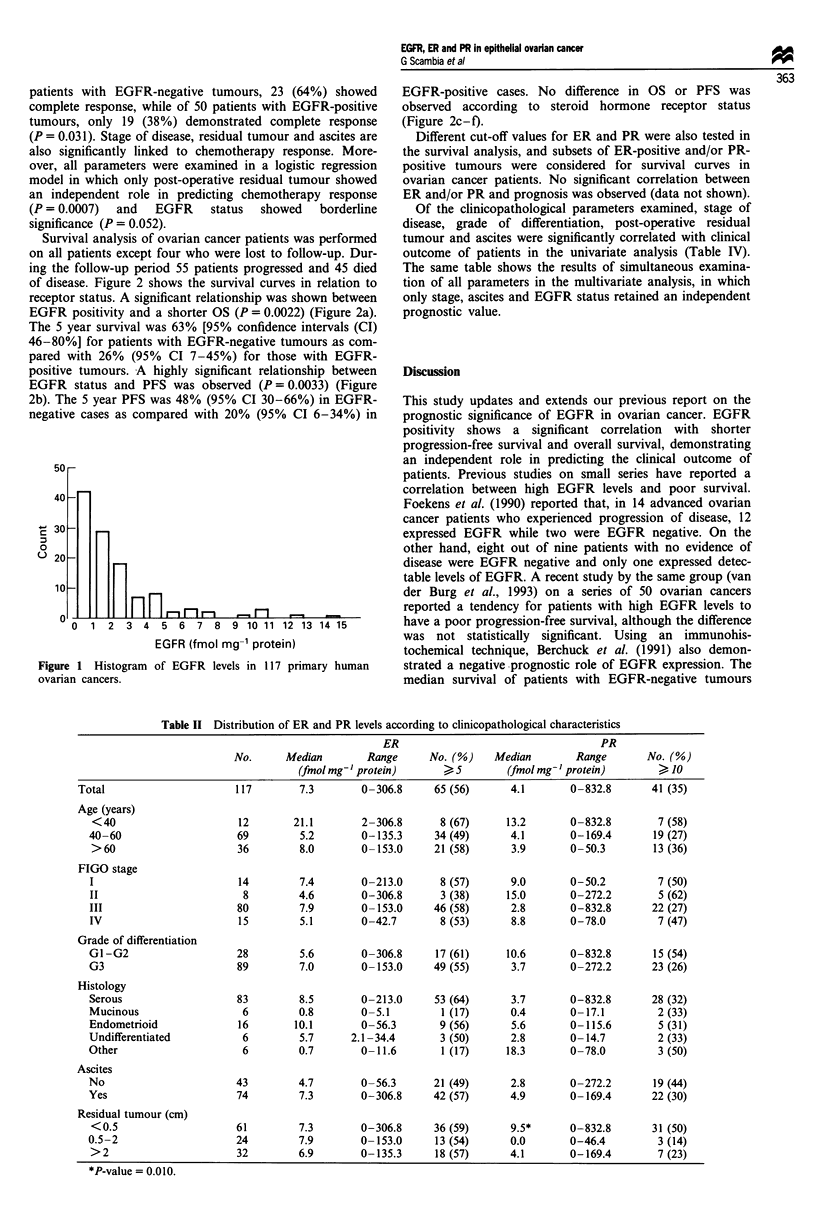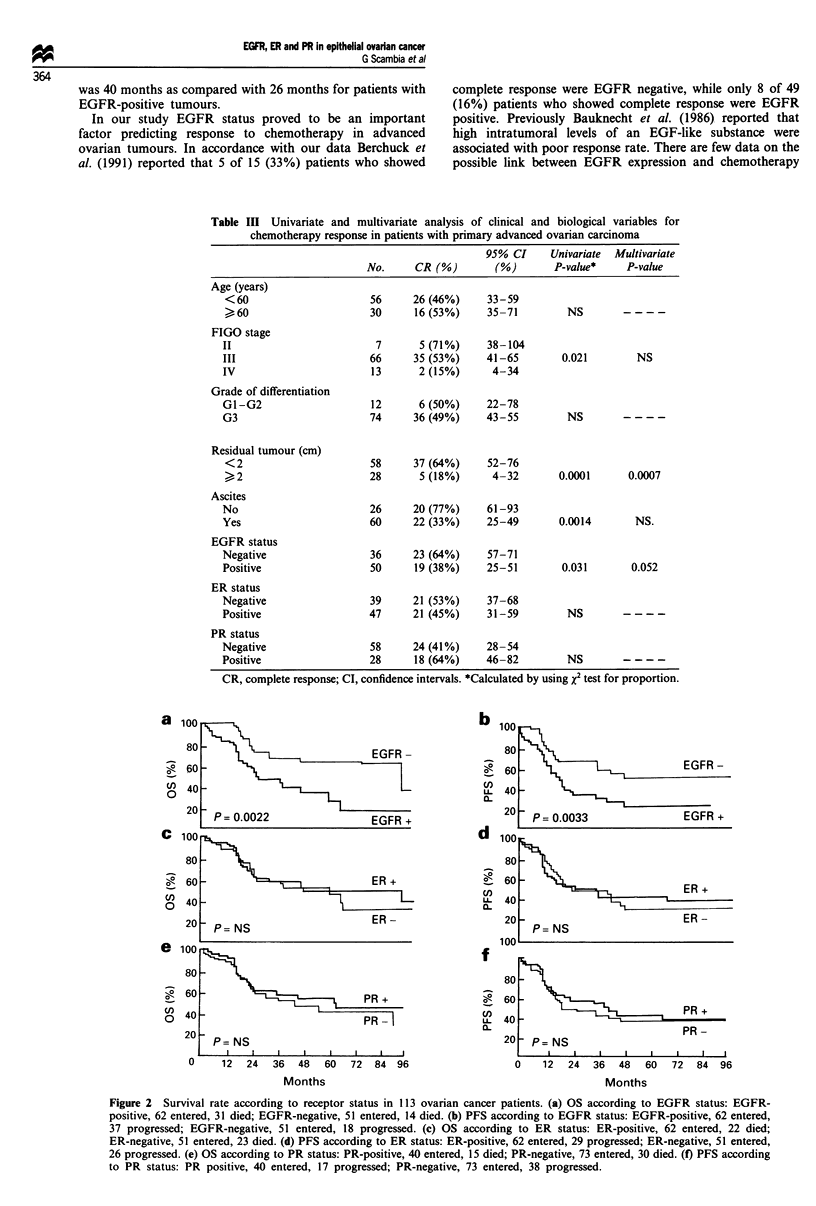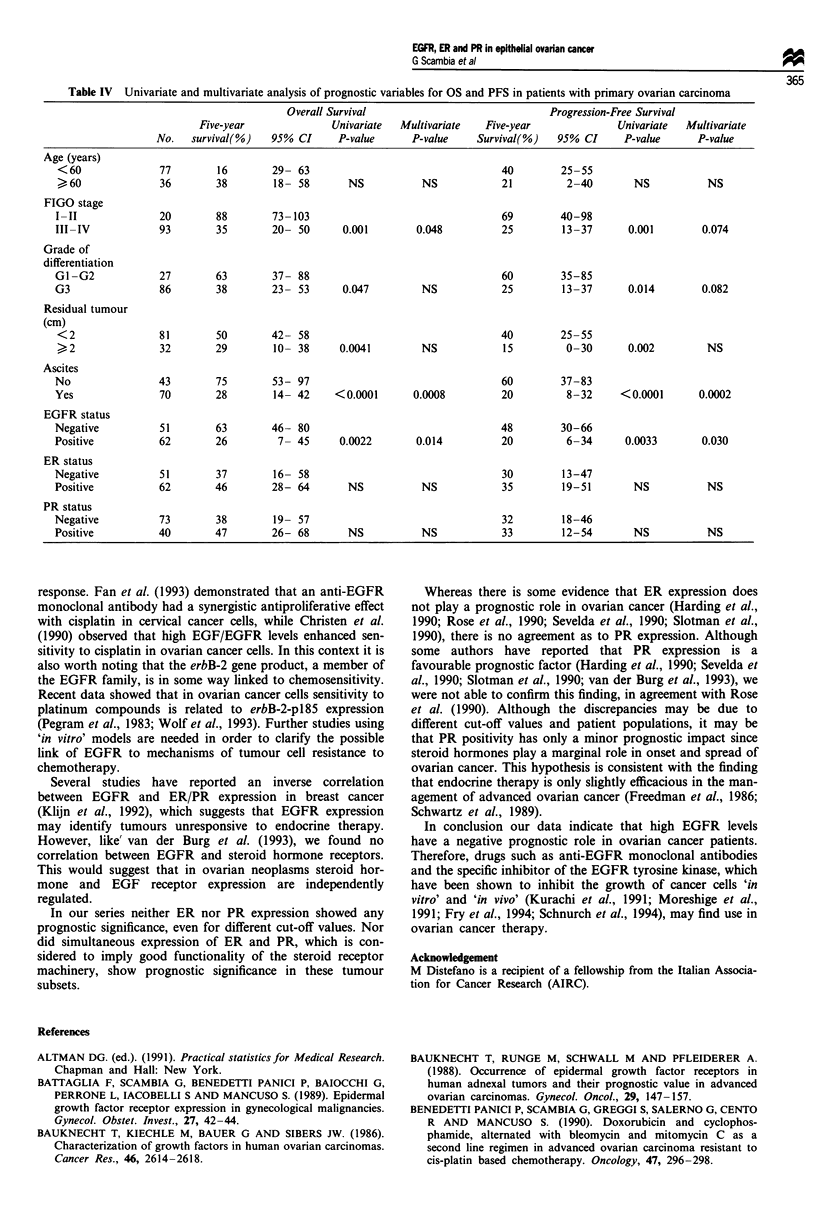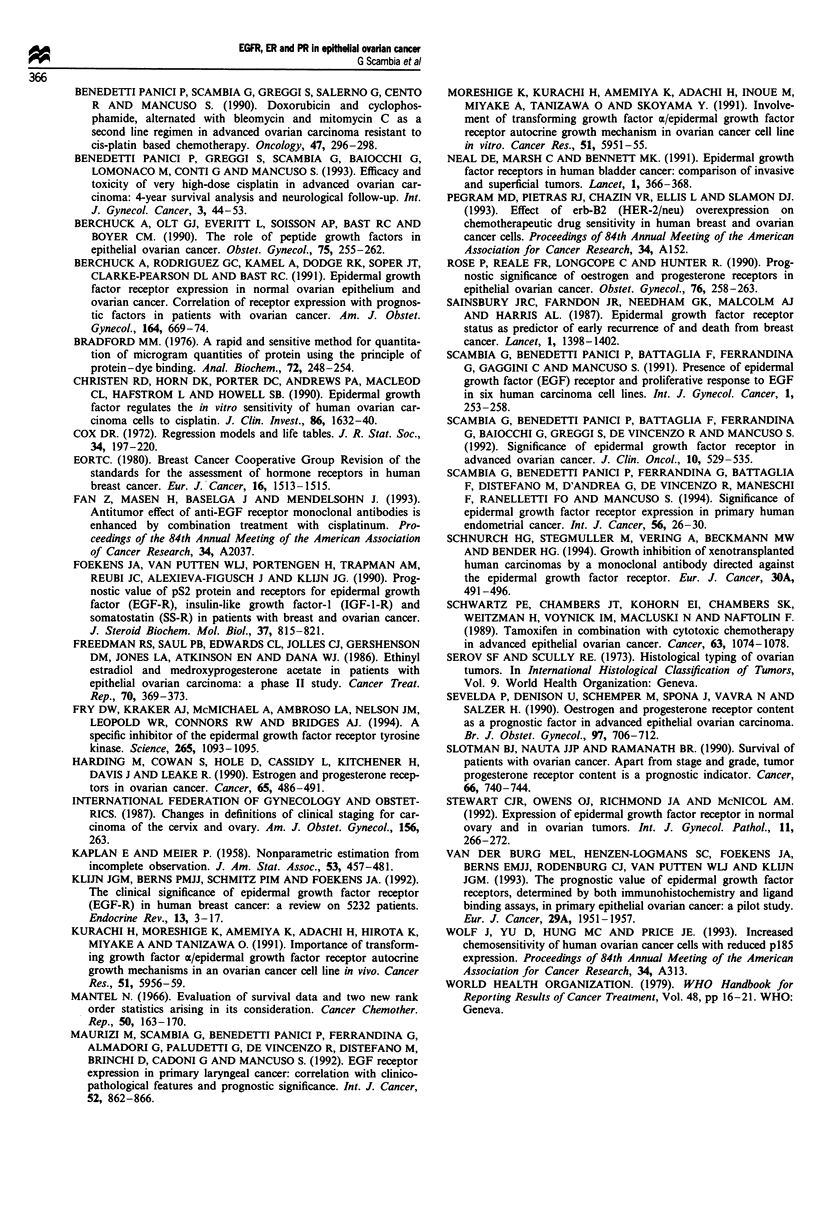# Epidermal growth factor, oestrogen and progesterone receptor expression in primary ovarian cancer: correlation with clinical outcome and response to chemotherapy.

**DOI:** 10.1038/bjc.1995.339

**Published:** 1995-08

**Authors:** G. Scambia, P. Benedetti-Panici, G. Ferrandina, M. Distefano, G. Salerno, M. E. Romanini, A. Fagotti, S. Mancuso

**Affiliations:** Department of Gynecology and Obstetrics, Catholic University, Rome, Italy.

## Abstract

The expression of epidermal growth factor receptor (EGFR), oestrogen receptor (ER) and progesterone receptor (PR) was assayed by a radioreceptor method in 117 primary ovarian cancers. EGFR was not significantly related to any of the clinicopathological parameters examined. In patients with stage II-IV disease who underwent second-look surgery after primary chemotherapy, a significant correlation between high EGFR levels and poor response to chemotherapy was demonstrated (P = 0.031). Moreover, post-operative residual tumour showed an independent role in predicting chemotherapy response (P = 0.0007) and EGFR status showed a borderline significance (P = 0.052) in the multivariate analysis. No correlation between steroid hormone receptors and clinicopathological parameters was observed. Whereas a significant relationship was shown between EGFR positivity and a shorter overall survival (OS) (P = 0.0022) and progression-free survival (PFS) (P = 0.0033), patient survival was not related to steroid hormone receptor status. Among the parameters tested only stage, ascites and EGFR status retained an independent prognostic value in the multivariate analysis.


British Journal of Cancer (1995) 72, 361-366
© 1995 Stockton Press All rights reserved 0007-0920/95 $12.00

### Epidermal growth factor, oestrogen and progesterone receptor expression in primary ovarian cancer: correlation with clinical outcome and response to chemotherapy

G Scambia, P Benedetti-Panici, G Ferrandina, M Distefano, G Salerno, ME Romanini, A Fagotti and S Mancuso

*Department of Gynecology and Obstetrics, Catholic University, Rome, Italy.*

**Summary** The expression of epidermal growth factor receptor (EGFR), oestrogen receptor (ER) and progesterone receptor (PR) was assayed by a radioreceptor method in 117 primary ovarian cancers. EGFR was not significantly related to any of the clinicopathological parameters examined. In patients with stage II-IV disease who underwent second-look surgery after primary chemotherapy, a significant correlation between high EGFR levels and poor response to chemotherapy was demonstrated ($P = 0.031$). Moreover, post-operative residual tumour showed an independent role in predicting chemotherapy response ($P = 0.0007$) and EGFR status showed a borderline significance ($P = 0.052$) in the multivariate analysis. No correlation between steroid hormone receptors and clinicopathological parameters was observed. Whereas a significant relationship was shown between EGFR positivity and a shorter overall survival (OS) ($P = 0.0022$) and progression-free survival (PFS) ($P = 0.0033$), patient survival was not related to steroid hormone receptor status. Among the parameters tested only stage, ascites and EGFR status retained an independent prognostic value in the multivariate analysis.

**Keywords:** epidermal growth factor receptor; oestrogen receptor; progesterone receptor; ovarian cancer

Experimental evidence has shown that the EGF/EGFR system is involved in ovarian cancer cell growth regulation (Berchuck *et al.*, 1990; Scambia *et al.*, 1991). At present, although EGFR expression has been widely demonstrated in ovarian cancer (Bauknecht *et al.*, 1988; Battaglia *et al.*, 1989; Berchuck *et al.*, 1991; Stewart *et al.*, 1992; van der Burg *et al.*, 1993), few studies have investigated the prognostic significance of EGFR (Bauknecht *et al.*, 1988; Berchuck *et al.*, 1991; van der Burg *et al.*, 1993). Some evidence does, however, demonstrate that high EGFR levels may be a negative prognostic indicator in many tumour types (Sainsbury *et al.*, 1987; Neal *et al.*, 1991; Maurizi *et al.*, 1992; Scambia *et al.*, 1994). Moreover, in breast cancer EGFR expression seems to be a feature of hormone-independent aggressive clinical behaviour (Klijn *et al.*, 1992). Our previous study (Scambia *et al.*, 1992) on a series of advanced ovarian carcinomas demonstrated higher EGFR levels in omental metastases than in primary tumours. We also reported that high EGFR levels were significantly associated with a greater risk of progression.

Data on the role of the expression of oestrogen receptor (ER) and progesterone receptor (PR) in ovarian cancer are discordant, although many authors have demonstrated that positivity for steroid hormone receptors is associated with a better prognosis (Harding *et al.*, 1990; Rose *et al.*, 1990; Sevelda *et al.*, 1990; Slotman *et al.*, 1990). In this report the prognostic significance of EGFR, ER and PR was simultaneously analysed in a large prospective series of primary ovarian cancer patients observed for a long follow-up period.

### Patients and methods

Our study included 117 primary ovarian cancer patients admitted to the Department of Gynecology of the Catholic University of Rome. All patients were staged according to the FIGO (1987) (International Federation of Gynecology and Obstetrics) classification. The World Health Organization (WHO) histological typing of ovarian tumours (Serov and Scully, 1973) was adopted. The clinicopathological features of the patients are listed in Table I. Chemotherapy was started 2-3 weeks after surgery. All patients received cisplatin-containing regimens (Benedetti Panici *et al.*, 1993).

Gynaecological examination, abdominopelvic ultrasonography, CA125 assay and radiological investigations, if necessary, were performed monthly for the clinical assessment of response, which was recorded according to WHO criteria (WHO, 1979). About 28 days after the last course, clinically complete responders underwent second-look laparoscopy. In laparoscopy-negative cases, second-look laparotomy was performed for the assessment of pathological response. During laparotomy and after peritoneal washings and careful inspection of the abdominal cavity, biopsy of all suspicious lesions was performed, and, in the case of no evidence of disease, at least 20 random biopsies were taken. Patients who initially had only an explorative laparotomy underwent a second laparotomy after chemotherapy, and a second cytoreduction was attempted. Pathologically complete responders received no further therapy, and all other patients were treated according to ongoing phase II studies (Benedetti Panici *et al.*, 1990).

### *Processing of tumour tissue*

Tissue specimens collected during primary surgery were frozen on dry ice shortly after surgical removal and stored at −80°C until processed. A representative section of specimens was retained for histopathological examination.

The membrane fraction and cytosol were prepared as described elsewhere (Scambia *et al.*, 1992). Briefly, tumour specimens were finely minced and homogenised in five volumes of ice-cold buffer consisting of 25 mM Tris, 1.5 mM EDTA, 5 mM sodium azide, 20% glycerol (TENG) plus 0.1% monothioglycerol, by applying several intermittent bursts of an Ultra-Turrax homogeniser. The crude homogenate was centrifuged at 7000 *g* for 20 min at 0°C in order to separate nuclear fraction from the cytosol fraction. The supernatants were then centrifuged at 105 000 *g* for 75 min at 0°C, obtaining the membrane pellet and the cytosolic fraction.

Correspondence: S Mancuso, Department of Gynecology and Obstetrics, Catholic University, Largo A. Gemelli, 8, 00168 Rome, Italy
Received 5 October 1994; revised 1 February 1995; accepted 7 February 1995

**Table I** Distribution of EGFR levels according to clinicopathological characteristics

| | No. | Median (fmol mg$^{-1}$ | Range protein) | No. ≥1.5 | (%) |
|---|---|---|---|---|---|
| Total | 117 | 1.5 | 0–14.8 | 63 | 54 |
| Age (years) | | | | | |
| <40 | 12 | 1.3 | 0–4.1 | 6 | 50 |
| 40–60 | 69 | 1.5 | 0–14.8 | 35 | 51 |
| >60 | 39 | 1.7 | 0–12.2 | 22 | 61 |
| FIGO stage | | | | | |
| I | 14 | 1.4 | 0–12.2 | 7 | 50 |
| II | 8 | 2.0 | 0–3.9 | 4 | 50 |
| III | 80 | 1.5 | 0–14.8 | 44 | 55 |
| IV | 15 | 1.8 | 0.3–10.5 | 9 | 60 |
| Grade of differentiation | | | | | |
| G1–G2 | 28 | 2.0 | 0–10.5 | 17 | 61 |
| G3 | 89 | 1.5 | 0–14.8 | 46 | 52 |
| Histology | | | | | |
| Serous | 83 | 1.5 | 0–14.8 | 45 | 54 |
| Mucinous | 6 | 2.7 | 0–5.3 | 4 | 67 |
| Endometrioid | 16 | 2.8 | 0–12.2 | 9 | 56 |
| Undifferentiated | 6 | 0.7 | 0–6.3 | 2 | 33 |
| Other | 6 | 3.3 | 0.9–10.2 | 4 | 67 |
| Ascites | | | | | |
| No | 43 | 1.1 | 0–12.2 | 16 | 37 |
| Yes | 74 | 1.9 | 0–10.5 | 49 | 66 |
| Residual tumour (cm) | | | | | |
| <0.5 | 61 | 1.5 | 0–14.8 | 33 | 53 |
| 0.5–2 | 24 | 1.5 | 0–4.3 | 14 | 58 |
| >2 | 32 | 2.2 | 0–10.5 | 18 | 57 |

*[$^{125}$I]EGF binding measurement*

The membrane pellet was resuspended in TENG plus 10 mM magnesium chloride. Aliquots of the purified suspension (100 µl containing 300–500 µg of protein) were incubated with [$^{125}$I]EGF (NEN Dupont De Nemours) (3.2 nM) in the presence or absence of unlabelled EGF (1 µM) for 16 h at room temperature in a final volume of 400 µl. Binding was stopped by the addition of 3 ml of TENG plus 0.1% bovine serum albumin. Pellets were obtained by centrifugation at 2000 *g* for 20 min at 0°C and counted in a gamma-counter for 1 min. Results were expressed as fmoles per mg of membrane protein (fmol mg$^{-1}$ protein).

In some cases Scatchard analysis of binding data was performed according to the previously described protocol (Scambia *et al.*, 1992). Dissociation constant ($K_d$) values ranged from 0.52 to 2.0 nmol l$^{-1}$. Protein concentration was measured by the Bradford method (1976). An EGFR level of 1.5 fmol mg$^{-1}$ protein was chosen as the cut-off value to define EGFR status.

*ER and PR measurement*

ER and PR were assayed with the dextran-coated charcoal (DCC) method according to the EORTC (1980) protocol and the cytosol fraction (1–2 mg protein ml$^{-1}$) was incubated (overnight at 4°C) with increasing concentrations of [$^3$H]oestradiol ([$^3$H]$E_2$) (sp.act. 120 Ci mmol$^{-1}$ from 0.05 nM to 1 nM) or [$^3$H]Organon 2058 (sp.act. 49 Ci mmol$^{-1}$ from 0.5 nM to 8 nM) (Amersham International) as radiolabelled compounds. ER and PR levels of 5.0 fmol mg$^{-1}$ protein and 10.0 fmol mg$^{-1}$ protein were arbitrarily chosen to define ER and PR status.

*Statistical analysis*

The Wilcoxon rank sum non-parametric test was used to analyse the relationship between ER, PR and EGFR expression and clinicopathological characteristics.

In order to normalise the variance of error the receptor data were transformed into $\log_{10}$ of data before performing Pearson's correlation test (Altman, 1991).

All medians and life tables were computed using the product-limit estimate of Kaplan and Meier (1958) and the curves were examined by means of the log-rank test (Mantel, 1966). Multivariate analysis was performed by the Cox proportional hazards model (Cox 1972). Progression-free survival (PFS) and overall survival (OS) were calculated from the date of first surgery to the date of clinical or pathological progression or death. The median follow-up was 19 months (range 2–110 months).

### Results

The distribution of EGFR levels in 117 primary ovarian tumours is shown in Figure 1. EGFR levels were skewed towards the lower values and ranged from 0 to 14.8 fmol mg$^{-1}$ protein, with a median value of 1.5 fmol mg$^{-1}$ protein. Sixty-three (54%) of the tumours were considered to be EGFR positive. Table I shows the distribution of EGFR levels according to clinicopathological characteristics of the patients. EGFR expression was not significantly related to any of the parameters examined.

ER ranged from 0 to 306.8 fmol mg$^{-1}$ protein (median 7.3 fmol mg$^{-1}$ protein) and PR ranged from 0 to 832.8 fmol mg$^{-1}$ protein (median 4.1 fmol mg$^{-1}$ protein). Using a cut-off of 5 and 10 fmol mg$^{-1}$ protein respectively, ER positivity was 56% and PR positivity was 35%. No correlation between steroid receptors and age, stage, grading, histotype or ascites was observed (Table II). PR expression was higher in patients with post-operative residual tumour <0.5 cm than in those with residual tumour ≥0.5 cm ($P = 0.01$). Using data transformed into logarithms, a direct correlation was found between ER and PR expression ($P = 0.017$), while no correlation was observed between EGFR and ER/PR distribution (data not shown).

Eighty-six patients with stage II–IV disease underwent second-look surgery after primary chemotherapy. Table III shows the univariate and multivariate analysis of clinical and biological variables for chemotherapy response in advanced ovarian carcinoma. There is a significant correlation between EGFR status and response to chemotherapy. In fact, of 36

patients with EGFR-negative tumours, 23 (64%) showed complete response, while of 50 patients with EGFR-positive tumours, only 19 (38%) demonstrated complete response ($P = 0.031$). Stage of disease, residual tumour and ascites are also significantly linked to chemotherapy response. Moreover, all parameters were examined in a logistic regression model in which only post-operative residual tumour showed an independent role in predicting chemotherapy response ($P = 0.0007$) and EGFR status showed borderline significance ($P = 0.052$).

Survival analysis of ovarian cancer patients was performed on all patients except four who were lost to follow-up. During the follow-up period 55 patients progressed and 45 died of disease. Figure 2 shows the survival curves in relation to receptor status. A significant relationship was shown between EGFR positivity and a shorter OS ($P = 0.0022$) (Figure 2a). The 5 year survival was 63% [95% confidence intervals (CI) 46–80%] for patients with EGFR-negative tumours as compared with 26% (95% CI 7–45%) for those with EGFR-positive tumours. A highly significant relationship between EGFR status and PFS was observed ($P = 0.0033$) (Figure 2b). The 5 year PFS was 48% (95% CI 30–66%) in EGFR-negative cases as compared with 20% (95% CI 6–34%) in

**Figure 1** Histogram of EGFR levels in 117 primary human ovarian cancers.

EGFR-positive cases. No difference in OS or PFS was observed according to steroid hormone receptor status (Figure 2c–f).

Different cut-off values for ER and PR were also tested in the survival analysis, and subsets of ER-positive and/or PR-positive tumours were considered for survival curves in ovarian cancer patients. No significant correlation between ER and/or PR and prognosis was observed (data not shown).

Of the clinicopathological parameters examined, stage of disease, grade of differentiation, post-operative residual tumour and ascites were significantly correlated with clinical outcome of patients in the univariate analysis (Table IV). The same table shows the results of simultaneous examination of all parameters in the multivariate analysis, in which only stage, ascites and EGFR status retained an independent prognostic value.

### Discussion

This study updates and extends our previous report on the prognostic significance of EGFR in ovarian cancer. EGFR positivity shows a significant correlation with shorter progression-free survival and overall survival, demonstrating an independent role in predicting the clinical outcome of patients. Previous studies on small series have reported a correlation between high EGFR levels and poor survival. Foekens *et al.* (1990) reported that, in 14 advanced ovarian cancer patients who experienced progression of disease, 12 expressed EGFR while two were EGFR negative. On the other hand, eight out of nine patients with no evidence of disease were EGFR negative and only one expressed detectable levels of EGFR. A recent study by the same group (van der Burg *et al.*, 1993) on a series of 50 ovarian cancers reported a tendency for patients with high EGFR levels to have a poor progression-free survival, although the difference was not statistically significant. Using an immunohistochemical technique, Berchuck *et al.* (1991) also demonstrated a negative prognostic role of EGFR expression. The median survival of patients with EGFR-negative tumours

**Table II** Distribution of ER and PR levels according to clinicopathological characteristics

| | No. | ER Median | ER Range (fmol mg$^{-1}$ protein) | No. (%) ≥5 | PR Median | PR Range (fmol mg$^{-1}$ protein) | No. (%) ≥10 |
|---|---|---|---|---|---|---|---|
| Total | 117 | 7.3 | 0–306.8 | 65 (56) | 4.1 | 0–832.8 | 41 (35) |
| Age (years) | | | | | | | |
| <40 | 12 | 21.1 | 2–306.8 | 8 (67) | 13.2 | 0–832.8 | 7 (58) |
| 40–60 | 69 | 5.2 | 0–135.3 | 34 (49) | 4.1 | 0–169.4 | 19 (27) |
| >60 | 36 | 8.0 | 0–153.0 | 21 (58) | 3.9 | 0–50.3 | 13 (36) |
| FIGO stage | | | | | | | |
| I | 14 | 7.4 | 0–213.0 | 8 (57) | 9.0 | 0–50.2 | 7 (50) |
| II | 8 | 4.6 | 0–306.8 | 3 (38) | 15.0 | 0–272.2 | 5 (62) |
| III | 80 | 7.9 | 0–153.0 | 46 (58) | 2.8 | 0–832.8 | 22 (27) |
| IV | 15 | 5.1 | 0–42.7 | 8 (53) | 8.8 | 0–78.0 | 7 (47) |
| Grade of differentiation | | | | | | | |
| G1–G2 | 28 | 5.6 | 0–306.8 | 17 (61) | 10.6 | 0–832.8 | 15 (54) |
| G3 | 89 | 7.0 | 0–153.0 | 49 (55) | 3.7 | 0–272.2 | 23 (26) |
| Histology | | | | | | | |
| Serous | 83 | 8.5 | 0–213.0 | 53 (64) | 3.7 | 0–832.8 | 28 (32) |
| Mucinous | 6 | 0.8 | 0–5.1 | 1 (17) | 0.4 | 0–17.1 | 2 (33) |
| Endometrioid | 16 | 10.1 | 0–56.3 | 9 (56) | 5.6 | 0–115.6 | 5 (31) |
| Undifferentiated | 6 | 5.7 | 2.1–34.4 | 3 (50) | 2.8 | 0–14.7 | 2 (33) |
| Other | 6 | 0.7 | 0–11.6 | 1 (17) | 18.3 | 0–78.0 | 3 (50) |
| Ascites | | | | | | | |
| No | 43 | 4.7 | 0–56.3 | 21 (49) | 2.8 | 0–272.2 | 19 (44) |
| Yes | 74 | 7.3 | 0–306.8 | 42 (57) | 4.9 | 0–169.4 | 22 (30) |
| Residual tumour (cm) | | | | | | | |
| <0.5 | 61 | 7.3 | 0–306.8 | 36 (59) | 9.5* | 0–832.8 | 31 (50) |
| 0.5–2 | 24 | 7.9 | 0–153.0 | 13 (54) | 0.0 | 0–46.4 | 3 (14) |
| >2 | 32 | 6.9 | 0–135.3 | 18 (57) | 4.1 | 0–169.4 | 7 (23) |

*$P$-value = 0.010.

was 40 months as compared with 26 months for patients with EGFR-positive tumours.

In our study EGFR status proved to be an important factor predicting response to chemotherapy in advanced ovarian tumours. In accordance with our data Berchuck *et al.* (1991) reported that 5 of 15 (33%) patients who showed complete response were EGFR negative, while only 8 of 49 (16%) patients who showed complete response were EGFR positive. Previously Bauknecht *et al.* (1986) reported that high intratumoral levels of an EGF-like substance were associated with poor response rate. There are few data on the possible link between EGFR expression and chemotherapy

**Table III** Univariate and multivariate analysis of clinical and biological variables for chemotherapy response in patients with primary advanced ovarian carcinoma

| | *No.* | *CR (%)* | *95% CI (%)* | *Univariate P-value** | *Multivariate P-value* |
|---|---|---|---|---|---|
| Age (years) | | | | | |
| <60 | 56 | 26 (46%) | 33–59 | | |
| ⩾60 | 30 | 16 (53%) | 35–71 | NS | ---- |
| FIGO stage | | | | | |
| II | 7 | 5 (71%) | 38–104 | | |
| III | 66 | 35 (53%) | 41–65 | 0.021 | NS |
| IV | 13 | 2 (15%) | 4–34 | | |
| Grade of differentiation | | | | | |
| G1–G2 | 12 | 6 (50%) | 22–78 | | |
| G3 | 74 | 36 (49%) | 43–55 | NS | ---- |
| Residual tumour (cm) | | | | | |
| <2 | 58 | 37 (64%) | 52–76 | | |
| ⩾2 | 28 | 5 (18%) | 4–32 | 0.0001 | 0.0007 |
| Ascites | | | | | |
| No | 26 | 20 (77%) | 61–93 | | |
| Yes | 60 | 22 (33%) | 25–49 | 0.0014 | NS. |
| EGFR status | | | | | |
| Negative | 36 | 23 (64%) | 57–71 | | |
| Positive | 50 | 19 (38%) | 25–51 | 0.031 | 0.052 |
| ER status | | | | | |
| Negative | 39 | 21 (53%) | 37–68 | | |
| Positive | 47 | 21 (45%) | 31–59 | NS | ---- |
| PR status | | | | | |
| Negative | 58 | 24 (41%) | 28–54 | | |
| Positive | 28 | 18 (64%) | 46–82 | NS | ---- |

CR, complete response; CI, confidence intervals. *Calculated by using $\chi^2$ test for proportion.

**Figure 2** Survival rate according to receptor status in 113 ovarian cancer patients. (**a**) OS according to EGFR status: EGFR-positive, 62 entered, 31 died; EGFR-negative, 51 entered, 14 died. (**b**) PFS according to EGFR status: EGFR-positive, 62 entered, 37 progressed; EGFR-negative, 51 entered, 18 progressed. (**c**) OS according to ER status: ER-positive, 62 entered, 22 died; ER-negative, 51 entered, 23 died. (**d**) PFS according to ER status: ER-positive, 62 entered, 29 progressed; ER-negative, 51 entered, 26 progressed. (**e**) OS according to PR status: PR-positive, 40 entered, 15 died; PR-negative, 73 entered, 30 died. (**f**) PFS according to PR status: PR positive, 40 entered, 17 progressed; PR-negative, 73 entered, 38 progressed.

**Table IV** Univariate and multivariate analysis of prognostic variables for OS and PFS in patients with primary ovarian carcinoma

| | | *Overall Survival* | | | | *Progression-Free Survival* | | | |
|---|---|---|---|---|---|---|---|---|---|
| | *No.* | *Five-year survival(%)* | *95% CI* | *Univariate P-value* | *Multivariate P-value* | *Five-year Survival(%)* | *95% CI* | *Univariate P-value* | *Multivariate P-value* |
| Age (years) | | | | | | | | | |
| <60 | 77 | 16 | 29- 63 | | | 40 | 25-55 | | |
| ⩾60 | 36 | 38 | 18- 58 | NS | NS | 21 | 2-40 | NS | NS |
| FIGO stage | | | | | | | | | |
| I-II | 20 | 88 | 73-103 | | | 69 | 40-98 | | |
| III-IV | 93 | 35 | 20- 50 | 0.001 | 0.048 | 25 | 13-37 | 0.001 | 0.074 |
| Grade of differentiation | | | | | | | | | |
| G1-G2 | 27 | 63 | 37- 88 | | | 60 | 35-85 | | |
| G3 | 86 | 38 | 23- 53 | 0.047 | NS | 25 | 13-37 | 0.014 | 0.082 |
| Residual tumour (cm) | | | | | | | | | |
| <2 | 81 | 50 | 42- 58 | | | 40 | 25-55 | | |
| ⩾2 | 32 | 29 | 10- 38 | 0.0041 | NS | 15 | 0-30 | 0.002 | NS |
| Ascites | | | | | | | | | |
| No | 43 | 75 | 53- 97 | | | 60 | 37-83 | | |
| Yes | 70 | 28 | 14- 42 | <0.0001 | 0.0008 | 20 | 8-32 | <0.0001 | 0.0002 |
| EGFR status | | | | | | | | | |
| Negative | 51 | 63 | 46- 80 | | | 48 | 30-66 | | |
| Positive | 62 | 26 | 7- 45 | 0.0022 | 0.014 | 20 | 6-34 | 0.0033 | 0.030 |
| ER status | | | | | | | | | |
| Negative | 51 | 37 | 16- 58 | | | 30 | 13-47 | | |
| Positive | 62 | 46 | 28- 64 | NS | NS | 35 | 19-51 | NS | NS |
| PR status | | | | | | | | | |
| Negative | 73 | 38 | 19- 57 | | | 32 | 18-46 | | |
| Positive | 40 | 47 | 26- 68 | NS | NS | 33 | 12-54 | NS | NS |

response. Fan *et al.* (1993) demonstrated that an anti-EGFR monoclonal antibody had a synergistic antiproliferative effect with cisplatin in cervical cancer cells, while Christen *et al.* (1990) observed that high EGF/EGFR levels enhanced sensitivity to cisplatin in ovarian cancer cells. In this context it is also worth noting that the *erb*B-2 gene product, a member of the EGFR family, is in some way linked to chemosensitivity. Recent data showed that in ovarian cancer cells sensitivity to platinum compounds is related to *erb*B-2-p185 expression (Pegram *et al.*, 1983; Wolf *et al.*, 1993). Further studies using '*in vitro*' models are needed in order to clarify the possible link of EGFR to mechanisms of tumour cell resistance to chemotherapy.

Several studies have reported an inverse correlation between EGFR and ER/PR expression in breast cancer (Klijn *et al.*, 1992), which suggests that EGFR expression may identify tumours unresponsive to endocrine therapy. However, like' van der Burg *et al.* (1993), we found no correlation between EGFR and steroid hormone receptors. This would suggest that in ovarian neoplasms steroid hormone and EGF receptor expression are independently regulated.

In our series neither ER nor PR expression showed any prognostic significance, even for different cut-off values. Nor did simultaneous expression of ER and PR, which is considered to imply good functionality of the steroid receptor machinery, show prognostic significance in these tumour subsets.

Whereas there is some evidence that ER expression does not play a prognostic role in ovarian cancer (Harding *et al.*, 1990; Rose *et al.*, 1990; Sevelda *et al.*, 1990; Slotman *et al.*, 1990), there is no agreement as to PR expression. Although some authors have reported that PR expression is a favourable prognostic factor (Harding *et al.*, 1990; Sevelda *et al.*, 1990; Slotman *et al.*, 1990; van der Burg *et al.*, 1993), we were not able to confirm this finding, in agreement with Rose *et al.* (1990). Although the discrepancies may be due to different cut-off values and patient populations, it may be that PR positivity has only a minor prognostic impact since steroid hormones play a marginal role in onset and spread of ovarian cancer. This hypothesis is consistent with the finding that endocrine therapy is only slightly efficacious in the management of advanced ovarian cancer (Freedman *et al.*, 1986; Schwartz *et al.*, 1989).

In conclusion our data indicate that high EGFR levels have a negative prognostic role in ovarian cancer patients. Therefore, drugs such as anti-EGFR monoclonal antibodies and the specific inhibitor of the EGFR tyrosine kinase, which have been shown to inhibit the growth of cancer cells '*in vitro*' and '*in vivo*' (Kurachi *et al.*, 1991; Moreshige *et al.*, 1991; Fry *et al.*, 1994; Schnurch *et al.*, 1994), may find use in ovarian cancer therapy.

**Acknowledgement**
M Distefano is a recipient of a fellowship from the Italian Association for Cancer Research (AIRC).

**References**

ALTMAN DG. (ed.). (1991). *Practical statistics for Medical Research*. Chapman and Hall: New York.

BATTAGLIA F, SCAMBIA G, BENEDETTI PANICI P, BAIOCCHI G, PERRONE L, IACOBELLI S AND MANCUSO S. (1989). Epidermal growth factor receptor expression in gynecological malignancies. *Gynecol. Obstet. Invest.*, **27**, 42-44.

BAUKNECHT T, KIECHLE M, BAUER G AND SIBERS JW. (1986). Characterization of growth factors in human ovarian carcinomas. *Cancer Res.*, **46**, 2614-2618.

BAUKNECHT T, RUNGE M, SCHWALL M AND PFLEIDERER A. (1988). Occurrence of epidermal growth factor receptors in human adnexal tumors and their prognostic value in advanced ovarian carcinomas. *Gynecol. Oncol.*, **29**, 147-157.

BENEDETTI PANICI P, SCAMBIA G, GREGGI S, SALERNO G, CENTO R AND MANCUSO S. (1990). Doxorubicin and cyclophosphamide, alternated with bleomycin and mitomycin C as a second line regimen in advanced ovarian carcinoma resistant to cis-platin based chemotherapy. *Oncology*, **47**, 296-298.

BENEDETTI PANICI P, SCAMBIA G, GREGGI S, SALERNO G, CENTO R AND MANCUSO S. (1990). Doxorubicin and cyclophosphamide, alternated with bleomycin and mitomycin C as a second line regimen in advanced ovarian carcinoma resistant to cis-platin based chemotherapy. *Oncology*, **47**, 296–298.

BENEDETTI PANICI P, GREGGI S, SCAMBIA G, BAIOCCHI G, LOMONACO M, CONTI G AND MANCUSO S. (1993). Efficacy and toxicity of very high-dose cisplatin in advanced ovarian carcinoma: 4-year survival analysis and neurological follow-up. *Int. J. Gynecol. Cancer*, **3**, 44–53.

BERCHUCK A, OLT GJ, EVERITT L, SOISSON AP, BAST RC AND BOYER CM. (1990). The role of peptide growth factors in epithelial ovarian cancer. *Obstet. Gynecol.*, **75**, 255–262.

BERCHUCK A, RODRIGUEZ GC, KAMEL A, DODGE RK, SOPER JT, CLARKE-PEARSON DL AND BAST RC. (1991). Epidermal growth factor receptor expression in normal ovarian epithelium and ovarian cancer. Correlation of receptor expression with prognostic factors in patients with ovarian cancer. *Am. J. Obstet. Gynecol.*, **164**, 669–74.

BRADFORD MM. (1976). A rapid and sensitive method for quantitation of microgram quantities of protein using the principle of protein-dye binding. *Anal. Biochem.*, **72**, 248–254.

CHRISTEN RD, HORN DK, PORTER DC, ANDREWS PA, MACLEOD CL, HAFSTROM L AND HOWELL SB. (1990). Epidermal growth factor regulates the *in vitro* sensitivity of human ovarian carcinoma cells to cisplatin. *J. Clin. Invest.*, **86**, 1632–40.

COX DR. (1972). Regression models and life tables. *J. R. Stat. Soc.*, **34**, 197–220.

EORTC. (1980). Breast Cancer Cooperative Group Revision of the standards for the assessment of hormone receptors in human breast cancer. *Eur. J. Cancer*, **16**, 1513–1515.

FAN Z, MASEN H, BASELGA J AND MENDELSOHN J. (1993). Antitumor effect of anti-EGF receptor monoclonal antibodies is enhanced by combination treatment with cisplatinum. *Proceedings of the 84th Annual Meeting of the American Association of Cancer Research*, **34**, A2037.

FOEKENS JA, VAN PUTTEN WLJ, PORTENGEN H, TRAPMAN AM, REUBI JC, ALEXIEVA-FIGUSCH J AND KLIJN JG. (1990). Prognostic value of pS2 protein and receptors for epidermal growth factor (EGF-R), insulin-like growth factor-1 (IGF-1-R) and somatostatin (SS-R) in patients with breast and ovarian cancer. *J. Steroid Biochem. Mol. Biol.*, **37**, 815–821.

FREEDMAN RS, SAUL PB, EDWARDS CL, JOLLES CJ, GERSHENSON DM, JONES LA, ATKINSON EN AND DANA WJ. (1986). Ethinyl estradiol and medroxyprogesterone acetate in patients with epithelial ovarian carcinoma: a phase II study. *Cancer Treat. Rep.*, **70**, 369–373.

FRY DW, KRAKER AJ, McMICHAEL A, AMBROSO LA, NELSON JM, LEOPOLD WR, CONNORS RW AND BRIDGES AJ. (1994). A specific inhibitor of the epidermal growth factor receptor tyrosine kinase. *Science*, **265**, 1093–1095.

HARDING M, COWAN S, HOLE D, CASSIDY L, KITCHENER H, DAVIS J AND LEAKE R. (1990). Estrogen and progesterone receptors in ovarian cancer. *Cancer*, **65**, 486–491.

INTERNATIONAL FEDERATION OF GYNECOLOGY AND OBSTETRICS. (1987). Changes in definitions of clinical staging for carcinoma of the cervix and ovary. *Am. J. Obstet. Gynecol.*, **156**, 263.

KAPLAN E AND MEIER P. (1958). Nonparametric estimation from incomplete observation. *J. Am. Stat. Assoc.*, **53**, 457–481.

KLIJN JGM, BERNS PMJJ, SCHMITZ PIM AND FOEKENS JA. (1992). The clinical significance of epidermal growth factor receptor (EGF-R) in human breast cancer: a review on 5232 patients. *Endocrine Rev.*, **13**, 3–17.

KURACHI H, MORESHIGE K, AMEMIYA K, ADACHI H, HIROTA K, MIYAKE A AND TANIZAWA O. (1991). Importance of transforming growth factor α/epidermal growth factor receptor autocrine growth mechanisms in an ovarian cancer cell line *in vivo*. *Cancer Res.*, **51**, 5956–59.

MANTEL N. (1966). Evaluation of survival data and two new rank order statistics arising in its consideration. *Cancer Chemother. Rep.*, **50**, 163–170.

MAURIZI M, SCAMBIA G, BENEDETTI PANICI P, FERRANDINA G, ALMADORI G, PALUDETTI G, DE VINCENZO R, DISTEFANO M, BRINCHI D, CADONI G AND MANCUSO S. (1992). EGF receptor expression in primary laryngeal cancer: correlation with clinicopathological features and prognostic significance. *Int. J. Cancer*, **52**, 862–866.

MORESHIGE K, KURACHI H, AMEMIYA K, ADACHI H, INOUE M, MIYAKE A, TANIZAWA O AND SKOYAMA Y. (1991). Involvement of transforming growth factor α/epidermal growth factor receptor autocrine growth mechanism in ovarian cancer cell line *in vitro*. *Cancer Res.*, **51**, 5951–55.

NEAL DE, MARSH C AND BENNETT MK. (1991). Epidermal growth factor receptors in human bladder cancer: comparison of invasive and superficial tumors. *Lancet*, **1**, 366–368.

PEGRAM MD, PIETRAS RJ, CHAZIN VR, ELLIS L AND SLAMON DJ. (1993). Effect of erb-B2 (HER-2/neu) overexpression on chemotherapeutic drug sensitivity in human breast and ovarian cancer cells. *Proceedings of 84th Annual Meeting of the American Association for Cancer Research*, **34**, A152.

ROSE P, REALE FR, LONGCOPE C AND HUNTER R. (1990). Prognostic significance of oestrogen and progesterone receptors in epithelial ovarian cancer. *Obstet. Gynecol.*, **76**, 258–263.

SAINSBURY JRC, FARNDON JR, NEEDHAM GK, MALCOLM AJ AND HARRIS AL. (1987). Epidermal growth factor receptor status as predictor of early recurrence of and death from breast cancer. *Lancet*, **1**, 1398–1402.

SCAMBIA G, BENEDETTI PANICI P, BATTAGLIA F, FERRANDINA G, GAGGINI C AND MANCUSO S. (1991). Presence of epidermal growth factor (EGF) receptor and proliferative response to EGF in six human carcinoma cell lines. *Int. J. Gynecol. Cancer*, **1**, 253–258.

SCAMBIA G, BENEDETTI PANICI P, BATTAGLIA F, FERRANDINA G, BAIOCCHI G, GREGGI S, DE VINCENZO R AND MANCUSO S. (1992). Significance of epidermal growth factor receptor in advanced ovarian cancer. *J. Clin. Oncol.*, **10**, 529–535.

SCAMBIA G, BENEDETTI PANICI P, FERRANDINA G, BATTAGLIA F, DISTEFANO M, D'ANDREA G, DE VINCENZO R, MANESCHI F, RANELLETTI FO AND MANCUSO S. (1994). Significance of epidermal growth factor receptor expression in primary human endometrial cancer. *Int. J. Cancer*, **56**, 26–30.

SCHNURCH HG, STEGMULLER M, VERING A, BECKMANN MW AND BENDER HG. (1994). Growth inhibition of xenotransplanted human carcinomas by a monoclonal antibody directed against the epidermal growth factor receptor. *Eur. J. Cancer*, **30A**, 491–496.

SCHWARTZ PE, CHAMBERS JT, KOHORN EI, CHAMBERS SK, WEITZMAN H, VOYNICK IM, MACLUSKI N AND NAFTOLIN F. (1989). Tamoxifen in combination with cytotoxic chemotherapy in advanced epithelial ovarian cancer. *Cancer*, **63**, 1074–1078.

SEROV SF AND SCULLY RE. (1973). Histological typing of ovarian tumors. In *International Histological Classification of Tumors*, Vol. 9. World Health Organization: Geneva.

SEVELDA P, DENISON U, SCHEMPER M, SPONA J, VAVRA N AND SALZER H. (1990). Oestrogen and progesterone receptor content as a prognostic factor in advanced epithelial ovarian carcinoma. *Br. J. Obstet. Gynecol.*, **97**, 706–712.

SLOTMAN BJ, NAUTA JJP AND RAMANATH BR. (1990). Survival of patients with ovarian cancer. Apart from stage and grade, tumor progesterone receptor content is a prognostic indicator. *Cancer*, **66**, 740–744.

STEWART CJR, OWENS OJ, RICHMOND JA AND McNICOL AM. (1992). Expression of epidermal growth factor receptor in normal ovary and in ovarian tumors. *Int. J. Gynecol. Pathol.*, **11**, 266–272.

VAN DER BURG MEL, HENZEN-LOGMANS SC, FOEKENS JA, BERNS EMJJ, RODENBURG CJ, VAN PUTTEN WLJ AND KLIJN JGM. (1993). The prognostic value of epidermal growth factor receptors, determined by both immunohistochemistry and ligand binding assays, in primary epithelial ovarian cancer: a pilot study. *Eur. J. Cancer*, **29A**, 1951–1957.

WOLF J, YU D, HUNG MC AND PRICE JE. (1993). Increased chemosensitivity of human ovarian cancer cells with reduced p185 expression. *Proceedings of 84th Annual Meeting of the American Association for Cancer Research*, **34**, A313.

WORLD HEALTH ORGANIZATION. (1979). *WHO Handbook for Reporting Results of Cancer Treatment*, Vol. 48, pp 16–21. WHO: Geneva.